# A General Shear-Dependent Model for Thrombus Formation

**DOI:** 10.1371/journal.pcbi.1005291

**Published:** 2017-01-17

**Authors:** Alireza Yazdani, He Li, Jay D. Humphrey, George Em Karniadakis

**Affiliations:** 1 Division of Applied Mathematics, Brown University, Providence, Rhode Island, United States of America; 2 Department of Biomedical Engineering, Yale University, New Haven, Connecticut, United States of America; University of Pennsylvania, UNITED STATES

## Abstract

Modeling the transport, activation, and adhesion of platelets is crucial in predicting thrombus formation and growth following a thrombotic event in normal or pathological conditions. We propose a shear-dependent platelet adhesive model based on the Morse potential that is calibrated by existing *in* *vivo* and *in* *vitro* experimental data and can be used over a wide range of flow shear rates ($100<\dot{\gamma }<28,000\;s^{-1}$). We introduce an Eulerian-Lagrangian model where hemodynamics is solved on a fixed Eulerian grid, while platelets are tracked using a Lagrangian framework. A force coupling method is introduced for bidirectional coupling of platelet motion with blood flow. Further, we couple the calibrated platelet aggregation model with a tissue-factor/contact pathway coagulation cascade, representing the relevant biology of thrombin generation and the subsequent fibrin deposition. The range of shear rates covered by the proposed model encompass venous and arterial thrombosis, ranging from low-shear-rate conditions in abdominal aortic aneurysms and thoracic aortic dissections to thrombosis in stenotic arteries following plaque rupture, where local shear rates are extremely high.

## Author Summary

Hemostasis (thrombus formation) is the normal physiological response that prevents significant blood loss after vascular injury. The resulting clots can form under different flow conditions in the veins as well as the arteries. The excessive and undesirable formation of clots (i.e., thrombosis) in our circulatory system may lead to significant morbidity and mortality. Some of these pathologies are deep vein thrombosis and pulmonary embolism and atherothrombosis (thrombosis triggered by plaque rupture) in coronary arteries, to name a few. The process of clot formation and growth at a site on a blood vessel wall involves a number of simultaneous processes including: multiple chemical reactions in the coagulation cascade, species transport and platelet adhesion all of which are strongly influenced by the hydrodynamic forces. Numerical models for blood clotting normally focus on one of the processes under a specific flow condition. Here, we propose a general numerical model that encompass a wide range of hemodynamic conditions in the veins and arteries, with individual platelets and their adhesive dynamics included explicitly in the models. Further, we include the biochemistry of coagulation cascade, which is essential to modeling thrombus formation, and couple that to our platelet aggregation model. The simulation results—tested against three different experiments—demonstrate that the proposed model is effective in capturing the *in* *vivo* and *in* *vitro* experimental observations.

## Introduction

Platelets are fundamental to both hemostasis and thrombosis in many vascular diseases, including abdominal aortic aneurysm (AAA), thoracic aortic aneurysm and dissection (TAAD), and carotid atherosclerosis [1–3]. Normal platelets do not interact with the healthy artery wall. In cases of endothelial injury or exposure of extracellular matrix to blood flow, however, platelets can quickly activate and cover the injured area to stop bleeding. The initial adhesion of platelets on the thrombogenic area can be attributed to a variety of platelet membrane receptor-ligand interactions, such as glycoprotein Ib(GPIb)-V-IX with immobilized von Willebrand Factor (vWF), GPIIb-IIIa (*α*_IIb_*β*_3_) with vWF, GPVI with collagen, *α*_2_*β*_1_ with collagen, *α*_IIb_*β*_3_ with fibrinogen, and so on, depending on the nature of the lesion [4] and the local shear rate of blood flow [5–7]. At low shear rates ($\dot{\gamma }<1000\;s^{-1}$), platelets adhere to the thrombogenic area through different pathways, relying on the exposed extracellular matrix (ECM) proteins [4, 5, 8]. On the other hand, as shear rate increases, interactions between immobilized vWF and GPIb become exclusive in initializing platelet aggregation while other interactions are broken down due to high bond failure rates [9–11]. The reason that vWF-GPIb interactions persist at such high shear rates (≈ 25,000 *s*^−1^ shown in *in* *vitro* experiments [11]) is that the vWF proteins, which are normally in a coiled state, tend to extend several fold in high-shear environments. The conformational change of vWF exposes the repeating functional A-1 domains in multimeric vWF, leading to enhanced adhesive interactions between GPIb and vWF [12–15]. Recently, experiments showed that the effect of vWF multimer extension was more pronounced in elongational flows, like in stenotic arteries, than in pure shear flows in a straight vessel [14].

The exposure of the subendothelial matrix triggers coagulation, which involves a network of tightly regulated enzymatic reactions leading to the production of the enzyme thrombin. Thrombin activates platelets and creates fibrin monomers that polymerize into a fibrous gel that stabilizes the clot. Coagulation is believed to be initiated when tissue factor (TF) molecules embedded in the vessel wall are exposed by injury and bind plasma enzyme factor VIIa [16]. Platelet activation can be induced by direct contact of platelets with collagens exposed in the subendothelium, by the action of thrombin, or by exposure to a threshold level of adenosine diphosphate (ADP) and thromboxane-A2 (TxA2). A finite quantity of ADP and TxA2 is released by a platelet during a time interval following the platelet’s activation. Numerous models are proposed for the systems biology of coagulation cascade among which the Kuharsky and Fogelson [16] is considered the most comprehensive one as it takes into account plasma-phase, subendothelial-bound and platelet-bound enzymes and zymogens. An extended version of this model was introduced by Leiderman and Kuharsky [17] to incorporate the spatial variations, represented by a system of partial and ordinary differential equations for the reactive transport of the chemical species. In this work, to reduce the computational cost, we use a slightly reduced-order model of coagulation proposed by Anand *et* *al*. [18], which has the advantage of including both TF and contact pathways in plasma.

The above-mentioned platelet-wall interactions and coagulation occur in the presence of blood flow. Hemodynamics plays a key role in transporting the platelets to the thrombogenic area via advection and diffusion. Begent and Born [19] performed an *in* *vivo* study on the effect of blood flow rates (or equivalently shear rates) on thrombus formation in a venous flow. They discovered that thrombus growth in venules with diameters of 40 − 60*μm* reached a maximum at a blood flow velocity around 400*μm*/*s* due to the balance between the number of platelets transported to the injured sites and the shear stress on the surface of the growing thrombus. Transport of platelets and other proteins involved in thrombus formation (fibrinogen and plasminogen, among others) becomes particularly important in the pathological conditions of AAA and TAAD. For example, platelets and reactants flow into an AAA and initiate intraluminal thrombus at specific locations in the aneurysm bulge [20, 21]. Such intraluminal thrombus can affect the mechanical properties of the local vessel wall, leading to increased risk of aneurysm rupture [22]. In TAAD, however, clinical evidence suggests that a completely thrombosed false lumen within the dissection results in an improved prognosis whereas a partially thrombosed false lumen may render the wall more vulnerable to further dissection or rupture [23]. Whether a fully thrombosed TAAD is formed or not could be attributed to the hemodynamics in the false lumen.

Numerical models have been developed to study platelet activation, adhesion, and aggregation in both physiological and pathological conditions [17, 24–30]. Pivkin *et* *al*. [25] developed a platelet model based on the force coupling method (FCM) to simulate platelet aggregation in a circular vessel. This model reproduced the experimental results in [19] and explored the effect of flow pulsatility on thrombus formation. Xu *et* *al*. [26, 27] developed a 2D multiscale model to simulate thrombus formation at different stages. Kamada *et* *al*. [24] used spring models for a variety of ligand-receptor interactions between platelets to investigate effects of ligand-receptor deficiencies on thrombus formation at different shear rates. Mountrakis *et* *al*. [29] used a 2D immersed boundary model and simulated platelets and red blood cells (RBCs) in blood vessels with saccular-shaped aneurysms. Biasetti *et* *al*. [31] solved advection-diffusion-reaction for multiple biomolecules in the coagulation cascade in fusiform-shaped AAAs to predict the location of intraluminal thrombus formation. In a very recent work, Tosenberger *et* *al*. [30] investigate the interaction of blood flow, platelet aggregation and plasma coagulation using a hybrid dissipative particle dynamics-continuum model in a 2D channel. The flow of plasma with the suspending platelets are solved using dissipative particle dynamics, while the regulatory network of plasma coagulation is described by a system of partial differential equations. Although considerable work has been conducted to simulate the advective and diffusive motions of platelets and other blood components in arterial flows, most studies focused on simplified arterial geometries. Transport and aggregation of platelets in dissections and stenoses have not yet been well studied due to the complex geometries and varying mechanisms of platelet adhesion under different hemodynamic conditions.

Our main goal in this paper is to develop a phenomenological model for platelet-wall and platelet-platelet adhesion, whose strength depends on the local shear rate, to represent different adhesion mechanisms. We model platelets as rigid spherical particles using the Lagrangian description within the context of FCM [32], as adopted in [25], whereas the hemodynamics and chemical transport are obtained from the solution of the Navier-Stokes (NS) equations and advection-diffusion-reaction (ADR) equations on a fixed Eulerian grid, respectively. We present the calibration of parameters in Eq (10) based on carefully chosen experimental data from the literature, where the platelet aggregation process is mainly separated from the complex biochemistry of the coagulation cascade. More specifically, we use the *in* *vivo* experimental data of Begent and Born for venous thrombus formation in mice [19] to calibrate our model for low-shear-rate regimes, where platelet aggregation is induced by the release of ADP *in* *vivo* causing the formation of white thrombi. In the high-shear regime, we use the results reported by Westein *et* *al*. for stenotic microchannels [14], where the shear rates can reach as high as 8,000 *s*^−1^. Here, platelet aggregation is caused by perfusing whole blood over surfaces coated by vWF/fibrinogen. Further, we use the experimental results in [33] for the purpose of testing our platelet aggregation model in a stenotic channel coated with collagen where shear rates are as high as 15,000 *s*^−1^. In the second part of the paper, we use a detailed model for blood coagulation coupled with our platelet aggregation model to address thrombus formation in arteriole-sized vessels similar to the *in* *vitro* experiment of Shen *et* *al*. [34] Our simulations agree well with the wide range of experimental data considered, thus suggesting the effectiveness of the proposed approach in modeling thrombus formation in blood vessels having complex geometries and under a broad range of flow conditions.

## Materials and Methods

Platelet motion within a flow field and adhesion to a damaged surface are solved together by coupling a spectral/hp element method (SEM) [35] with a FCM [32]. Specifically, SEM is used to solve the flow field and the reactive transport of chemical species on a fixed Eulerian grid, whereas FCM is implemented to describe the two-way interactions between the blood flow and Lagrangian particles (*i*.*e*., platelets).

### Platelet transport and aggregation

Simulations with fully resolved RBC and platelet suspensions in blood are challenging due to the computational cost of modeling millions of particles. In order to reduce the computational cost, we take blood as a continuous medium, and the effect of RBCs on platelet margination is taken into account by assuming that blood flow at the inlet of the simulated vessels is fully developed and platelets are already marginated toward the vessel wall. We prescribe the distribution of the platelets at the inlets based on the reported experimental distributions of Yeh *et* *al*. [36]. The reported distributions are obtained for platelet-sized latex beads suspended in whole blood flowing in tubes with ≈ 200 *μm* diameter at 40% hematocrit, where the average wall shear rate is ≈ 500 *s*^−1^.

In FCM, the translational velocity of each platelet particle is estimated by the local average of the fluid velocity weighted by a Gaussian kernel function. In our simulations, we assume platelets to be spheres with radius of 1.5 *μm* and number density of 300,000 *mm*^−3^, while blood is assumed to be an incompressible Newtonian fluid. Applying the FCM method detailed in [32], the governing equations for the incompressible flow are

$$\rho \left(\frac{\partial u}{\partial t}+u\cdot \nabla u\right)=-\nabla p+\mu \nabla^{2}u+f\left(x,t\right)\;,$$ (1)

∇·u=0, (2)

$$f\left(x,t\right)=\sum_{n=1}^{N}F^{n}\;\Delta \left(x-Y^{n}\left(t\right)\right)\;,$$ (3)

where **u**, *p*, and *μ* are the flow velocity, pressure and blood viscosity, respectively, and **F**^*n*^ in Eq (3) is the force due to particle *n* (discussed later). The effect of the platelets on the flow field is incorporated into the body force term **f** (**x**, *t*) in the Navier-Stokes Eq (1). The contribution of each platelet whose center of mass is located at **Y**^*n*^ to the flow at position **x** is smoothed by a Gaussian distribution kernel Δ(**X** ≡ (**x** − **Y**^**n**^)), where Δ(**X**) is

$$\Delta \left(X\right)={\left(2\pi \sigma^{2}\right)}^{-3/2}\;\text{exp}\left(-X\cdot X/2\sigma^{2}\right)\;,$$ (4)

with *σ* the standard deviation of the kernel, which is related to particle radius *a* through $\sigma =a/\sqrt{\pi }$. The governing equations are written in weak form and the domain is discretized using spectral elements that allow high order Jacobi polynomials. Time integration is performed using a semi-implicit splitting scheme [35].

The velocity of each platelet **V**^*n*^ is calculated by interpolating the local flow velocity at the location of a platelet using the same Gaussian kernel of Eq (4) (different standard deviations may be used for force and velocity interpolations)

$$V^{n}=\frac{dY^{n}}{dt}=\int u\;\Delta \left(x-Y^{n}\left(t\right)\right)dx\;,$$ (5)

where the position vectors for all the platelets are updated at each time step using a second-order Euler forward scheme. As mentioned above, a near-wall excess distribution of platelets is imposed for platelets entering the domain to take into account the effect of margination. It is known that the lateral platelet diffusion is enhanced through its collisions with RBCs, which is on the order of *D*_*p*_ = 10^−7^ *cm*^2^/*s*. This value varies with local shear rates and hematocrit. In one of our sensitivity studies, we augment platelet transport through the following equation for the displacement

$$dY^{n}=V^{n}dt+{\left(2D_{p}dt\right)}^{1/2}\;R$$ (6)

where **R** is a Gaussian random variable with mean 0 and variance 1. The effective diffusion coefficient is taken to be a function of the local shear rate based on the equation proposed by Wootton *et* *al*. [37], $D_{p}=7\left(10^{-9}\right)\;\dot{\gamma }\;\left(cm^{2}/s\right)$, where the enhanced diffusion is considered in the lateral direction only.

The net force acting on each platelet **F**^*n*^ is written by

$$F^{n}=-\frac{4}{3}\pi r^{3}\left(\rho_{plat}-\rho_{fluid}\right)\frac{dV^{n}}{dt}+F_{inter}\;,$$ (7)

where the first term is the inertial force resulting from the density difference between the platelets and blood flow. The second term accounts for the interaction forces between platelets with each other and the wall, which represent overall effects of different ligand-receptor interactions.

We propose a phenomenological model based on Morse potential *U*_*Morse*_ to model the attractive/repulsive interactions between platelets, namely

$$U_{Morse}=D_{e}{\left[1-e^{-\beta d(r/d-1)}\right]}^{2}\;,$$ (8)

where *D*_*e*_ is the energy depth contributing to the strength of the interaction force and *β* controls the width of the energy well; *r* is the distance between the platelets centeroids and *d* = 3 *μm* is the equilibrium distance between two platelets and is selected to be the diameter of platelet.

**Fig 1:**
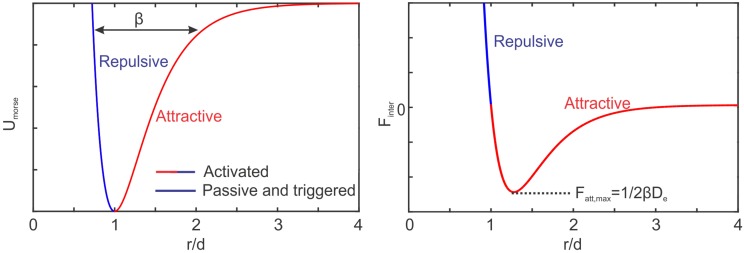
Schematic of Morse potential and the resulting adhesive force. Morse potential is used in this study to mimic inter-platelet attractive/repulsive forces. Passive and triggered platelets only generate repulsive forces to prevent overlap, whereas activated platelets attract each other as well.

As shown in Fig 1, the Morse potential is similar to a Lennard-Jones potential; it consists of both attractive (at *r* > *d*) and repulsive parts (at *r* < *d*). The Morse potential possesses a softer repulsive-core, however, which is much more stable when simulating platelet aggregation. The magnitude of the interaction forces resulting from this Morse potential can be obtained by taking the variation of the potential with respect to interparticle distance *r*, which gives

$$F_{inter}=-\frac{\partial U_{Morse}}{\partial r}=2D_{e}\beta \left[e^{-2\beta d(r/d-1)}-e^{-\beta d(r/d-1)}\right]\;.$$ (9)

The maximum attractive forces between the two platelets can be calculated by ∂*F*_*inter*_/∂*r* = 0, which gives *F*_*max*_ = *βD*_*e*_/2 occurring at (*r*/*d* − 1) = log(2)/*βd*. In our simulations, *βd* is selected to be 2.5 and thus the maximum attractive force is obtained at *r* ≈ 1.27*d*. The undetermined parameter *D*_*e*_, which mainly controls the magnitude of the platelet interaction forces, is determined from experimentally measured thrombus formation and growth under different hemodynamic conditions. Toward this end, platelets are assumed to exist in three different states, namely *passive*, *triggered*, or *activated*. In passive or triggered states, platelets are non-adhesive, hence only repulsive forces are applied between them to prevent cellular overlap as shown by the blue line segment in Fig 1. If a passive platelet interacts with an activated platelet, it becomes triggered and will switch to an activated state after an activation delay time *τ*_*act*_. When two activated platelets interact with each other, a repulsive force results when *r* < *d* and an attractive force when *r* > *d* as shown by the red line segment in Fig 1. For calibrating our platelet aggregation model, we consider an interaction distance of 2*d* between platelets within which resting platelets can get activated. It should be noted that in the *in* *vitro* experiments for platelet aggregation, platelets can bind directly to the collagen or vWF-coated surfaces without activation. This may be followed by irreversible platelet activation and the release of ADP, whereas thrombin production is excluded from these experiments.

Next, we present a phenomenological model that correlates the adhesion force to the local shear rate. The correlation has to be able to cover different flow conditions (*e*.*g*., clotting in venules vs. arteries) and adhesive mechanisms (*e*.*g*., adhesion at low vs. high shear rates). For that purpose we propose a shear-dependent correlation for *D*_*e*_ following a hyperbolic tangent formula

$$D_{e}\left(\lambda_{2}\right)=D_{e}^{h}\;\left[\text{tanh}\left(\frac{\lambda_{2}-\lambda_{2}^{l}}{1000}\right)+\frac{D_{e}^{l}}{D_{e}^{h}}+1\right]\;,$$ (10)

where $\lambda_{2}=\sqrt{2\;D:D}$ is the square root of the second invariant of the fluid strain-rate tensor **D**, $D_{e}^{l}$ and $D_{e}^{h}$ determine the adhesive forces at low and high shear rates, respectively, and $\lambda_{2}^{l}$ is the shear rate threshold value where transition from low to high shear regime takes place.

**Fig 2:**
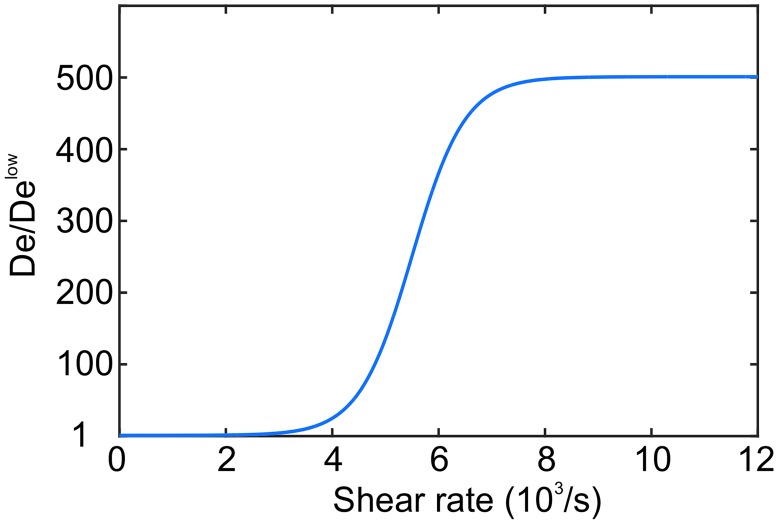
Plot of the Morse potential’s well depth *D*_*e*_. Following Eq (10), *D*_*e*_ is calibrated as a function of *λ*_2_, the second invariant of the flow strain rate tensor, where $D_{e}^{low}$ defines the lower bound of the platelet’s adhesive force.

The constants in Eq (10) are calibrated using *in* *vivo* and *in* *vitro* experiments, which results in the function plotted in Fig 2. The model is tested for clotting in venules at low shear rates and microfluidic devices with a constriction resembling atherosclerosis plaques, which can induce high shear rates of the order of ≈ 20,000 *s*^−1^ [11]. We determined the constants to be $D_{e}^{h}=500D_{e}^{l}$, where $D_{e}^{l}\approx 2.1\times 10^{-17}\;Nm$ and $\lambda_{2}^{l}=5,500\;s^{-1}$. The calibrated values are both inspired by the available data from the recent study by Mehrabadi *et* *al*. [11], which presents a predictive model for high-shear thrombus growth, and by the observations from our numerical simulations mimicking the *in* *vivo* and *in* *vitro* experiments. Additional details will be discussed in section Results.

### Coagulation cascade

In this section, we briefly describe the mathematical model for the combined TF and contact pathway of blood coagulation originally proposed by Anand *et* *al*. [18]. More details of the model as well as the reaction rate constants and parameters are given in the SI Text. The model takes into account plasma-phase enzymes and zymogens, and coagulation inhibitors, where the advection-diffusion-reaction (ADR) equations for plasma-phase enzymes, zymogens or complexes lead to a system of 20 partial differential equations (PDEs) in the following form

$$\frac{\partial c_{i}}{\partial t}+u\cdot \nabla c_{i}=D_{i}\nabla^{2}c_{i}+S_{i}\;\;i=1\text{ to }20,$$ (11)

where *c*_*i*_ and *D*_*i*_ are the concentration and diffusion coefficient for each reactant, respectively, and *S*_*i*_ represents the rate of production or destruction of that reactant. A zero-flux boundary condition is imposed for most reactants in the ADR equations except for a few reactants (factors IX/IXa and X/Xa) to initiate the coagulation, which is in the form: −*D*_*i*_∂*c*_*i*_/∂*n* = *B*_*i*_, where *n* is the unit normal on the boundary and *B*_*i*_ is the related surface reaction. For zymogens, the upstream concentrations at the inlet and initial concentrations are set to their normal plasma values, whereas all enzyme and complex concentrations are initially set to a very small nonzero value.

Passive platelets can directly bind to the collagen on the subendothelium and become activated. They also become activated by exposure to sufficiently high concentrations of thrombin, TxA2 and ADP. We define an activation function *ω*(**x**, *t*) = [IIa]/[IIa]_*thr*_ + [ADP]/[ADP]_*thr*_ + [TxA2]/[TxA2]_*thr*_, where the subscript “*thr*” corresponds to the threshold concentration that activates the platelets. Platelets transporting through the regions with values of *ω* > 1 will become activated. In this study, we assume a negligible activating effect for TxA2, and the threshold values of [IIa]_*thr*_ = 1 *nM* [38] and [ADP]_*thr*_ = 1,000 *nM* [39].

Experimental measurements show that platelets release a finite quantity of ADP to the blood stream within 5 seconds following activation [40]. Here, we assign a normal distribution for the release function $R\left(t\right)=\text{exp}\left[-{\left(t-\mu \right)}^{2}/\sigma_{r}^{2}\right]\;/\;\sqrt{2\pi \sigma_{r}^{2}}$ with the mean release time of *μ* = 3 *s* and variance of $\sigma_{r}^{2}=2\;s^{2}$. We use the same FCM Gaussian kernel function to evaluate the spatial distribution of ADP release from each platelet

$$S_{rel}\left(x,t\right)=\sum_{n=1}^{N}A^{\prime }\;R\left(t-t_{act}^{n}\right)\;\Delta \left(x-Y^{n}\left(t\right)\right)\;,$$ (12)

where *A*′ = 3 × 10^−8^ *nM* is the ADP content for each platelet [39], and $t_{act}^{n}$ is the time at which platelet *n* becomes activated.

Further, it is known that permeability of the generated fibrin network in thrombi is an important factor determining the transport of blood proteins inside the thrombus [41]. To couple the porosity of fibrin network to the local flow field we introduce a Brinkman term in the form of − (*μ*/*k*) **u** to the right hand side of the NS Eq (1), where *μ* is the blood viscosity and *k* is permeability inside the fibrin network, and is considered to be locally varying with the concentration of fibrin. The experimental measurements of Kim *et* *al*. showed an inverse power law permeability with respect to the fibrin volume fraction [41]. Assuming that the same correlation exists with respect to the fibrin concentration [Ia], we write *k* as

$$k=8{\left(10\right)}^{-12}{\left(\frac{\left[\text{Ia}\right]}{{\left[\text{Ia}\right]}_{thr}}\right)}^{-1.8}\left(m^{2}\right)\;,$$ (13)

where [Ia]_*thr*_ = 5,000 *nM* is the threshold concentration at the core of the clot causing the lowest clot permeability *k* = 8(10)^−12^ *m*^2^.

To initiate and drive the coagulation, a spatially varied concentration level of subendothelium-bound TF-VIIa complex is prescribed at the site of injury. This results in a few reactions at the wall (represented by flux conditions) that form enzymes IXa and Xa that drive the TF pathway. The concentration of [TF-VIIa]^0^ was initially set at 0.25 for venous flows, which is in the range of concentration levels in the numerical study of Kuharsky and Fogelson [16] (estimated to be initially O(1)nM at shear rate 500 *s*^−1^). The concentration of TF-VIIa complex decreases with increasing concentration of fibrin as there will be less binding sites on the vessel subendothelium for the complex. The exact correlation for the variation of [TF-VIIa] is not known, and thus, we assume a cubic function in the form of [TF-VIIa] = [TF-VIIa]^0^(1 − ([Ia]/[Ia]_*thr*_)^3^).

## Results

### Clotting at low shear rates

As introduced above, platelet adhesion and aggregation in blood flow at low shear rates (< 1,000 *s*^−1^) may stimulate multiple ligand-receptor interactions, depending on the exposed ECM proteins (but is not strongly dependent on GPIb-vWF binding). We assume that the overall effect of interactions between receptors and ligands is incorporated into the adhesive model of Eqs (9) and (10), with $D_{e}^{l}$ the undetermined parameter.

**Fig 3:**
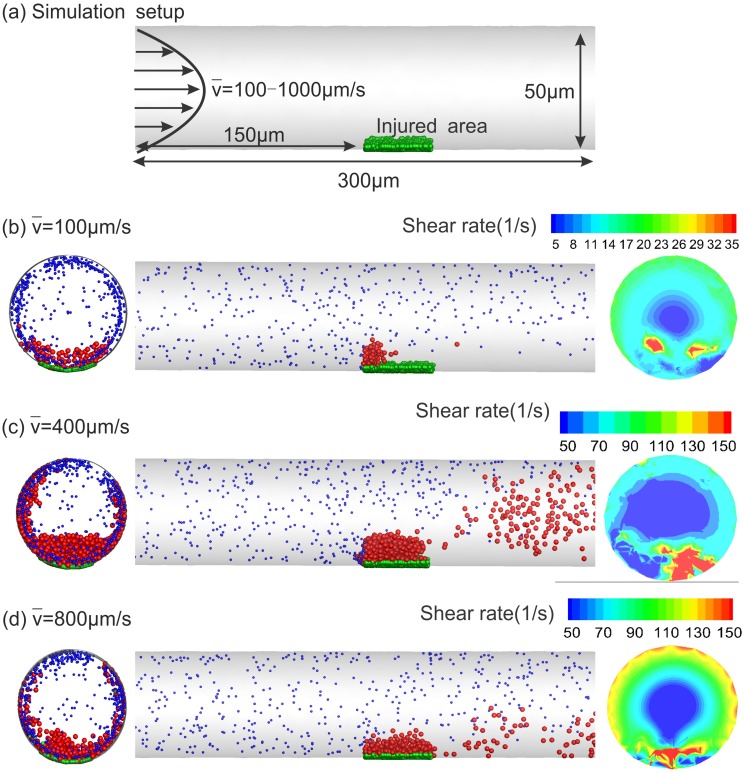
Snapshots of low-shear blood clotting in a 50*μm* circular tube. (a) schematic of the simulation setup; (b), (c) and (d) velocity at the inlet is parabolic with the mean velocity equal to 100, 400, 800 *μm*/*s*, respectively. Green particles represent the seeded platelets at the site of injury, whereas blue and red particles are passive and activated platelets, respectively. Blue particles are plotted smaller for clarity. Activated particles can form thrombus and adhere to the injured wall. The circular plots on the left column present the side views of each tube showing the clot that is formed by activated platelets, whereas the circular plots on the right column are cross-sections taken at the center of clots showing the contours of *λ*_2_ on those planes.

First, we consider venous thrombus formation and growth similar to the *in* *vivo* experiment of Begent and Born [19]. The geometry consists of a straight tube of 50*μm* diameter and 300*μm* length as shown in Fig 3a. A parabolic velocity profile is imposed at the inlet with variable average velocities in the range of 100 − 1,000 *μm*/*s*, which result in a maximum Reynolds number *Re* ≈ 0.02, whereas a zero-Neumann velocity boundary condition is imposed at the outlet. To mimic the site of injury and initiate platelet aggregation, we place fixed activated particles (green particles in Fig 3a) uniformly at the bottom of the channel 150 − 180 *μm* from the inlet. These fixed infinitesimal particles only interact with moving platelets in the blood flow without interfering with the flow field. Fresh platelets (red particles) are inserted at the inlet proportional to the local flow rate with a density of 300,000*mm*^−3^, and are removed from the system once they exit the channel.

The snapshots of the developed thrombi are given in Fig 3b–3d for several flow rates, where red particles represent activated platelets that can adhere to the site of injury and blue particles are resting platelets. We also plot *λ*_2_ contours on the circular cross-sections located at the middle of clots in Fig 3b–3d. The contours clearly show the elevated shear rates on the thrombus surface upon increasing blood velocity, which lead to disaggregation at higher blood velocities.

**Fig 4:**
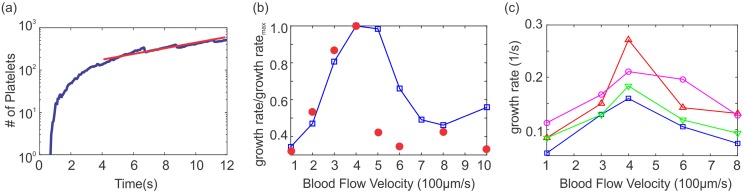
Low-shear simulation results of blood clotting in a 50*μm* circular tube. (a) A typical example of the number of platelets aggregated in the thrombus vs. time, plotted in semi-log axes. Exponential growth is achieved after a few seconds. The exponential growth rate is computed by fitting the data (red line). (b) Exponential growth rates (normalized by the maximum value) computed from the simulations and plotted as a function of blood flow velocity (−□−). Here, the size of injury is 30*μm* and platelet concentration is taken as 300,000*mm*^−3^; experimental data extracted from Begent and Born [19] (○). (c) Exponential growth rates derived from simulations for three different conditions: platelet concentration taken as 500,000*mm*^−3^ (−△−); increased size of injury to 60*μm* (−▽−); and the inclusion of shear-induced platelet’s drift according to Eq (6) (−○−). Results from (b) replotted here for comparison (−□−).

The number of platelets in the aggregate at the injured area is recorded for a period of 10 seconds, from which we can calculate the aggregate growth rate. A representative thrombus growth rate is plotted in Fig 4a on a semi-log axes, which shows an initial transient followed by a steady exponential growth of the form ∼exp(*α*_*g*_*t*), similar to *in* *vivo* observations of Begent and Born. After fitting the numerical data, we are able to extract the exponential growth rate *α*_*g*_ for different blood flow velocities, which were then normalized by the maximum growth rate and plotted in Fig 4b. Note that, at a lower blood velocity 100 *μm*/*s*, aggregation occurs slowly due to the smaller number of platelets transported to the injured region. As blood velocity increases to 400 *μm*/*s*, more platelets are delivered to the injured region, contributing to faster growth rate. If blood velocity is increased further to 800 *μm*/*s*, the higher shear stresses on the surface of the platelet aggregate limit further aggregation, and thus reduces the growth rate. Similar non-monotonic trends can be observed in the experimental data of Begent and Born, which are extracted from their article and plotted in Fig 4b for comparison. Similarly, Tosenberger *et* *al*. [30] observed non-monotone dependence of clot growth rate followed by the clot detachment upon increasing the shear rate. Our numerical values for exponential growth rates are close to the results in Pivkin *et* *al*. and [25] Kamada *et* *al*. [24], although the magnitude of the exponential growth rates from experiment is several fold higher than from the simulation. There could be a few reasons for this discrepancy, including the mismatch in the size of the injury site and the difference in normal platelet concentration between *in* *vivo* experiments and our simulations. We looked at this problem more closely by separately increasing the size of injury to 60*μm* or increasing the platelet density in our simulations to 500,000*mm*^−3^. These additional results are shown in Fig 4(c) along with the original results of Fig 4b. We observe similar trends in all three curves. The effect of increasing the size of injury marginally affects the exponential growth rates, whereas the increase in platelet density increases the exponential growth rates more notably. Another process that could potentially affect the growth rates is the shear-induced platelet drift toward the wall or the thrombus. Although in our numerical scheme particles are inserted close to the walls as suggested by the experimental observations, the particles may be subjected to further margination as they pass through the narrower vessel at the site of injury. We tested this hypothesis by using an empirical equation for enhanced diffusion of platelets at higher shear rates proposed by Wootton *et* *al*. [37] (see Eq (6)). The results are plotted in Fig 4(C), which indeed show an increase in the growth rates by 50%.

By adjusting the interaction forces between the platelet particles, we were able to reproduce the dependence of the growth rate on blood velocity reported in [19]. The resulting maximum attractive force applied in the simulation is found to be *F*_*adh*,*max*_ ≈ 10*pN* corresponding to $D_{e}^{l}\approx 2.1\times 10^{-17}\;Nm$.

### Platelet aggregation at high shear rates

In atherosclerotic arteries, the presence of plaques generates fluid mechanical conditions that promote high-shear platelet aggregation and thrombus formation [14, 15]. Nesbitt *et* *al*. [15] observed that platelet aggregation was predominately in the post-stenosis region and proposed that the aggregation of platelets was resulted from platelet tethering. Westein *et* *al*. [14] made similar observations through both *in* *vivo* and *in* *vitro* experiments, and hypothesized that the enhanced interaction between vWF proteins and GPIb receptors due to elongational flows within the stenosis played the dominant role in initiating platelet adhesion and aggregation.

**Fig 5:**
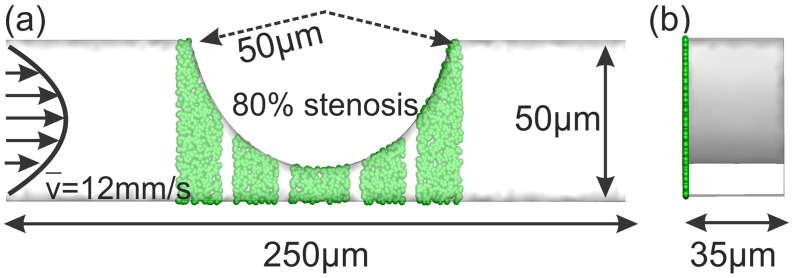
Schematic of a microchannel with constriction representing a stenosis and used for modeling platelet aggregation at high shear rate. (a) view normal to the flow direction; (b) side view along the flow direction; green particles are seeded uniformly on the left wall to represent vWF-coated regions similar to the experimental device in Westein *et* *al*. [14].

In order to estimate platelet interaction forces that cause platelet aggregation at elevated shear rates, we first use the data of Westein *et* *al*. from a microfluidic device with different degrees of stenosis. A schematic of the simulation domain is shown in Fig 5, where the channel height is 50*μm* and its depth is 35*μm*. We consider four different occlusion levels of 20, 40, 60, and 80%. The mean flow velocity at the channel inlet is set as ${\bar{u}}_{inlet}=12\;mm/s$, equivalent to *Re* ≈ 0.5 and an inlet wall shear rate ${\dot{\gamma }}_{w}=1,000\;s^{-1}$ consistent with the microchannel experiment [14]. Fixed and activated particles (green particles in Fig 5) representing vWF, are placed on discontinuous strips on the lower side of the channel wall.

**Fig 6:**
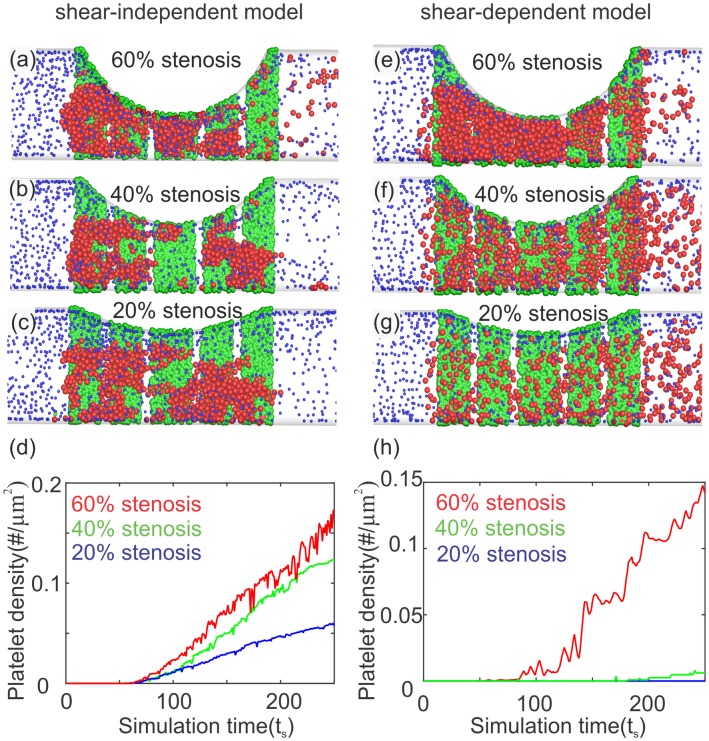
Simulation results for platelet aggregation at high shear rates with occlusion levels of 20–60% corresponding to the undisturbed maximum wall shear rates 2,000 − 6,000 *s*^−1^. A fixed value ($D_{e}=500D_{e}^{l}$) for platelet’s adhesive forces is used (a-d); shear-dependent correlation in Eq (10) is used (e-h). (a), (b) and (c) Snapshots of platelet aggregation inside 60, 40 and 20% stenoses, respectively. (e), (f) and (g) Snapshots of platelet aggregation inside 60, 40 and 20% stenoses, respectively. No aggregation is found for 20% stenosis; (d) and (h) density of adhered platelets inside the stenosis vs. simulation time. Color coding for particles is the same as in Fig 3. Here, the activation delay time is *τ*_*act*_ = 0*s*.

We performed numerical simulations for different occlusion levels to calibrate the platelet-wall and platelet-platelet adhesive forces, which suggested that an approximately two orders of magnitude higher adhesive force is required for platelet aggregation at such elevated shear rates. We plot snapshots of platelets aggregated in the channel at different occlusion levels in Fig 6. In the first column (Fig 6a–6c) we present results where the adhesive forces are increased uniformly (through $D_{e}^{h}=500D_{e}^{l}$), irrespective of the local shear rate magnitude. Based on these snapshots and their related curves for the density of aggregated platelets in Fig 6d, we observe that platelets aggregate inside the stenosis for all geometries and flow conditions, even at 20% occlusion where no aggregation was reported in the experiment of Westein *et* *al*. This nonphysical trend necessitates the use of a shear-dependent model for adhesive forces similar to Eq (10). Next, we present snapshots of platelet aggregation simulated using Eq (10) in the second column (Fig 6e–6g) along with their aggregate density curves in Fig 6h. Here, we observe a significant improvement in the results with no aggregation for 20% occlusion, a delayed aggregation for 40% occlusion, and a significant increase in the density of aggregated platelets for 60% stenosis. Altogether, these trends successfully capture the behavior observed in the experiment of Westein *et* *al*. [14]

**Fig 7:**
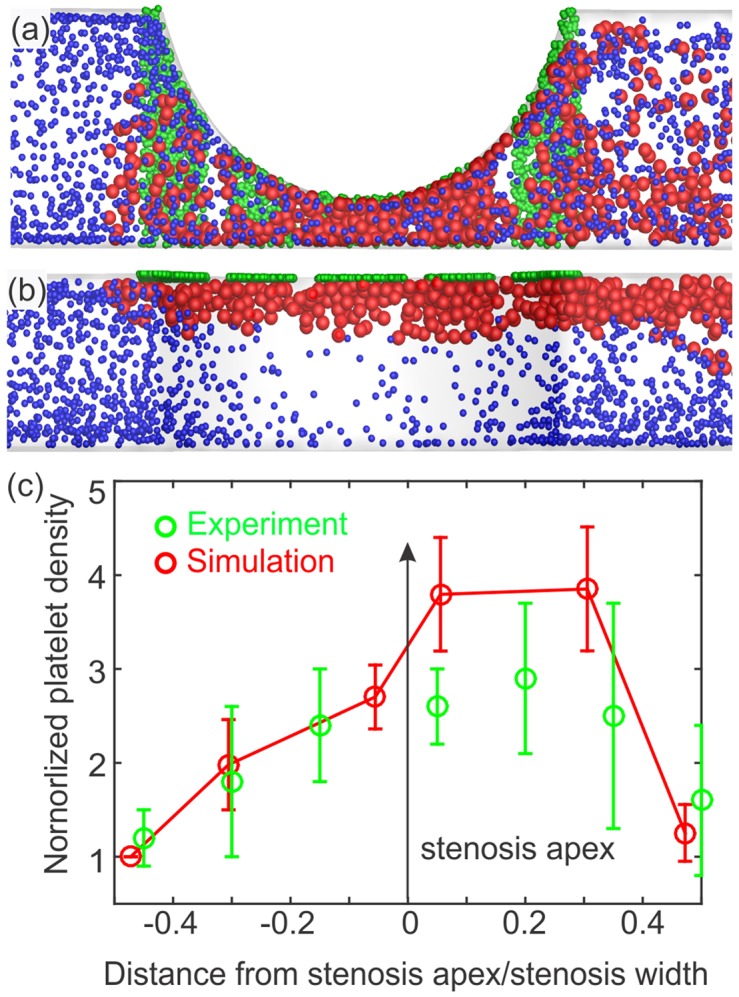
Simulation results for platelet aggregation at high shear rates inside an 80% stenosis, where the undisturbed maximum wall shear rate is 8,000 *s*^−1^. (a) View normal to the flow direction, and (b) view from above. Color coding for particles is the same as in Fig 3. (c) Normalized density of adhered platelets throughout the stenosis along the flow direction vs. normalized axial location. The density is normalized by the number of adhered platelets at the inlet, and axial distance is normalized by the length of the stenosis. Simulation results (−□−) are based on the activation delay time *τ*_*act*_ = 6 ± 3*ms*, and the error bars are computed based on 5 simulations with the same *τ*_*act*_; experimental data (−○−) are extracted from Westein *et* *al*. [14] and plotted for comparison.

One important trend in the *in* *vivo* experimental results of Westein *et* *al*. [15] is the enhanced platelet aggregation at the outlet of stenosis compared to its inlet. This effect may be attributed to several factors, including elongation of vWF multimers and enhanced diffusion of agonists at the outlet of the stenosis. To model these effects using the current numerical approach, we introduce a new parameter *τ*_*act*_ that delays the activation of platelets once stimulated by other activated platelets. This new parameter can be adjusted to control the distribution of aggregated platelets in the stenotic region. Either no or very short delay times will lead to aggregation at the inlet toward the middle parts of stenosis, whereas platelets with properly adjusted activation delays do not become adhesive until they pass the apex of the stenosis. We plot the results of platelet aggregation in an 80% stenosis in Fig 7 with both numerical and experimental platelet density profiles inside the stenosis. We assume that activation delay time is a random number with a uniform distribution and set as *τ*_*act*_ = 6 ± 3*ms* for each platelet. Fig 7b reveals that this model produces results similar to the experiment given a shear-dependent adhesive force and activation delay time.

#### Comparison with other experimental data

Other experimental studies, using both microfluidic devices [33, 42] and macroscopic-size glass stenosis devices [43, 44], similarly focused on the thrombus growth rate and occlusion time inside a stenosis. These results suggest that platelet aggregation on collagen coated walls is centered mostly at the stenosis apex where wall shear rates are the highest, but spreading to the inlet and outlet regions of the stenosis. The range of initial wall shear rates at the apex tested in the experiment of Li *et* *al*. [33] is wider than those of Westein *et* *al*., reaching as high as 13,000 *s*^−1^.

**Fig 8:**
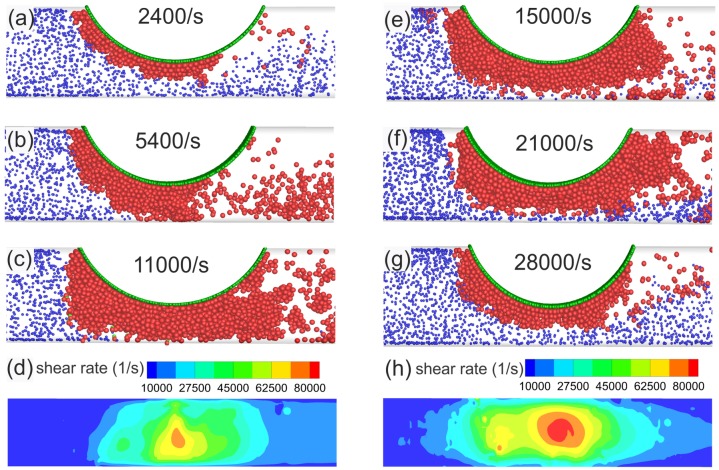
Simulation results for platelet aggregation at high shear rates in a fixed geometry with 60% stenosis, whereas the increase in flow rate develops high shear rates in the stenosis. Printed number in each figure is the undisturbed maximum wall shear rates encountered in each stenosis (before aggregation occurs). Full occlusion is observed in (b) and (c). Color coding for particles is the same as in Fig 3, and green particles are seeded on the circular arc only. Here, the activation delay time is *τ*_*act*_ = 0 *s*. (d) and (h) Wall shear rate contours plotted on the opposite wall of the arc for the simulations with undisturbed wall shear rate values of 15,000 and 28,000 *s*^−1^, respectively.

To test the performance of our proposed model, we use the simulation setup of Fig 5a with a 60% asymmetric stenosis. Fixed and activated platelets are seeded on the interior surface of the circular arc (green particles in Fig 8) to initiate platelet aggregation. The geometry remains fixed, while we test different flow rates to create a range of wall shear rates at the apex from 1,000 − 15,000 *s*^−1^. We plot snapshots of aggregated platelets on the stenotic wall taken at the same instant for different initial wall shear rates in Fig 8a–8f. We find no occlusion when shear rate is less than 2,400 *s*^−1^, comparable to 1,500 *s*^−1^ reported in microfluidic experimental results of Li *et* *al*. [33]. Full stenosis occlusion can be achieved when shear rate is elevated above 5,400 *s*^−1^, which is comparable to the threshold shear rate 4,000 *s*^−1^ reported by Li *et* *al*. We also find that upon increasing the shear rate from 15,000 to 28,000 *s*^−1^, parts of the formed aggregate mostly on the outer edge of thrombus start to detach as the shear forces increase dramatically and overcome adhesive forces (see Fig 8d–8f). Such embolic events are clearly important *in* *vivo*. Further, to show the magnitude of shear rate acting on the outer layer of thrombus in the stenosis, we plot the magnitude of wall shear rates on the wall opposite the curved face after thrombus is formed at 15,000 and 28,000*s*^−1^ in Fig 8g and 8h, respectively. As expected, the wall shear rate intensity increases by increasing the flow rate, thus leading to smaller thrombus size inside the stenosis in the case of 28,000 *s*^−1^.

### Modeling venous thrombus formation

**Fig 9:**
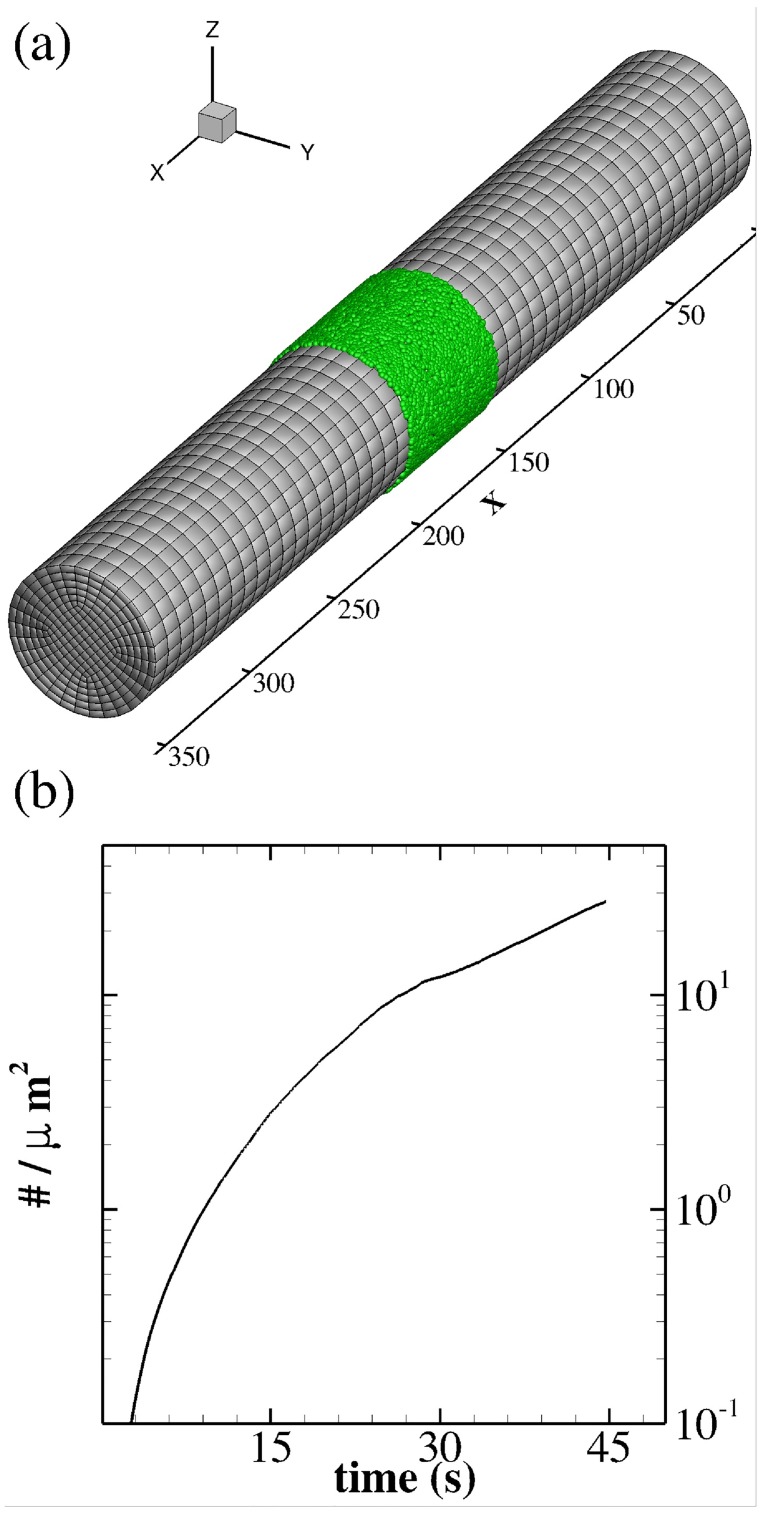
Blood coagulation and thrombus formation in a venous flow. (a) Schematic of the simulation setup with the seeded particles (green) placed circumferentially to represent the subendothelial matrix (150 − 200 *μm*). The hexahedral elements show the structured grid used to solve the N-S and ADR equations. (b) Time course of platelet aggregation density on the injured area at 64 *s*^−1^ shear rate.

Having the adhesion model calibrated for different flow conditions, we include the coagulation process in blood flowing over a site of injury that expresses tissue factor (TF), which is the primary stimulus for initiation of coagulation. We perform simulations in a circular tube of 50 *μm* diameter and 350 *μm* length representing a venule. A cylindrical patch with seeded platelets is placed in the middle of the tube to represent the site of injury, where thrombosis is allowed to initiate (see Fig 9a). We consider the lower range of flow rates that are normally seen in venous blood flows corresponding to a shear rate of 64 *s*^−1^. The time course of aggregate density is plotted in Fig 9b; it shows similar exponential growth after an initial transient time (≈ 5*s*), and in agreement with the experiments of Begent and Born [19].

**Fig 10:**
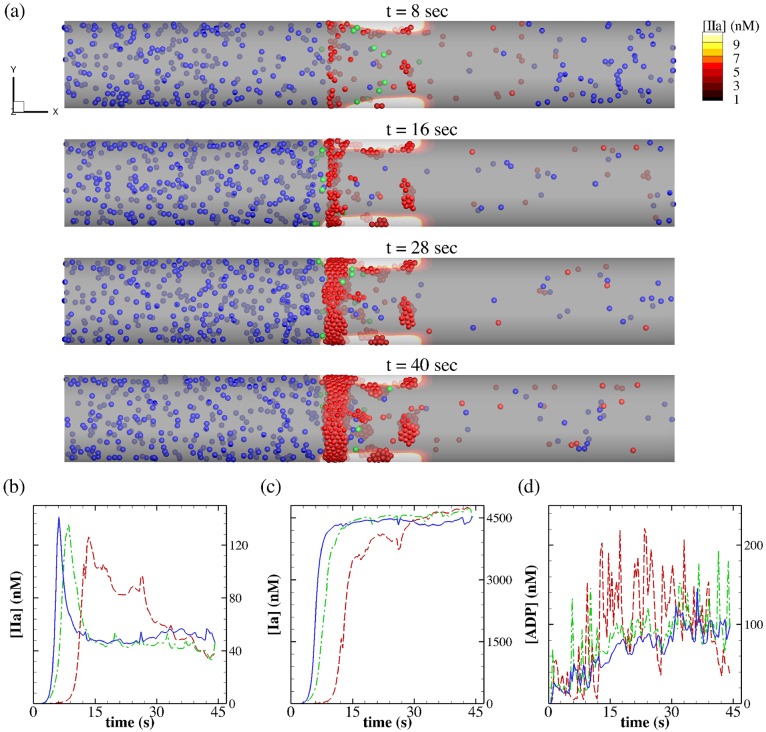
Blood coagulation and thrombus formation in a venous flow ($\dot{\gamma_{w}}=64\;s^{-1}$). (a) Snapshots of platelet aggregation at different time instants superposed on the contours of thrombin ([IIa]). Color coding for particles is the same as in Fig 3. (b), (c) and (d) Concentration profiles of thrombin ([IIa]), fibrin ([Ia]) and [ADP], respectively, at three axial positions on the site of injury: *x* = 157 *μm* (−−), *x* = 177 *μm* (− ⋅ −) and *x* = 193 *μm* (—).

Our initial numerical observations based on the kinetic rates taken from Anand *et* *al*. [18] showed negligible to no thrombin production. This signifies the effect of blood flow on the transport of coagulation reactants away from the site of injury before they can initiate the cascade. Only when we increase each reaction constant by approximately 10 fold, could we observe the production of thrombin mostly downstream of the injury site (see Fig 10a). As shown in the snapshots of Fig 10a, platelets can adhere directly to the exposed collagen and initially form aggregates independent from the coagulation process. As the aggregation grows both radially and axially, blood flow becomes stagnant at the site of aggregation, which in turn, reduces the advective transport of coagulation reactants away from the injury. This can be further seen in Fig 10b and 10c, where thrombin and fibrin concentration profiles are plotted at three different axial locations. The profiles show an almost independent thrombin burst and the subsequent fibrin generation at the center and downstream of the injury, whereas a delayed thrombin burst occurs at *t* ≈ 12 *s* proximal to the injury, where platelet aggregation is more pronounced. Further, the concentration profiles of ADP in Fig 10c show an increase as more platelets aggregate and release their granule including ADP. We observe significant oscillation in the concentration profile proximal to the injury as platelets activity and aggregate is higher in that region.

## Discussion

Platelet adhesion occurs via receptor-ligand bindings, but many different receptors and ligands are active under different shear conditions. Specifically, three shear rate regimes have been described: low shear “venous flow” (< 200 *s*^−1^), primarily governed by fibrinogen and the GPIIb-IIIa; intermediate shear “arterial flow” (500 − 4,000 *s*^−1^), primarily governed by GPIb, GPIIb-IIIa; and high shear “pathologic flow” (> 4,000 *s*^−1^) commonly found in diseased, constricted, or stenosed arteries, primarily governed by vWF and GPIb [42, 45]. The binding kinetics are thus diverse and for some integrins not very well characterized, thus inclusion of these details in numerical models will increase their uncertainty as well as the associated computational cost. In this study, our primary objective was to establish a phenomenological shear-dependent model for platelet adhesive dynamics based on the available experimental data for low [19], intermediate [14], and high shear flow [33] conditions. The various quantities reported in these experiments, such as thrombus shape and growth rate as well as platelet aggregate densities, enable us to tune our model for a wide range of shear rates.

We chose a Morse potential to generate the attractive/repulsive forces with a shear-dependent parameter *i*.*e*., the strength of the potential $D_{e}∼f\left(\dot{\gamma }\right)$, that is calibrated through Eq (10) for different flow conditions. The repulsive forces rise exponentially for inter-platelet distances less than *r* < *d* to prevent cellular overlap. As mentioned in section Materials and Methods, we set the interaction range of the Morse potential *βd* = 2.5 so that the potential strength *D*_*e*_ is the only parameter left to be tuned. The adjusted interaction range implies that particles will not induce forces for distances *r* ⪆ 3*d* as shown in Fig 1. Further increase in *βd* is not physiologically correct as the potential and adhesive forces become long-range. Although the present adhesive potential is not capable of directly addressing the kinetics of bond formation/dissociation, it can capture different binding phenomena implicitly due to the effect of local flow conditions and shear rates. The transport velocity of a platelet moving close to the vessel wall is proportional to ${\dot{\gamma }}_{w}$ meaning that at low shear rates the change in the inter-particle distance *r* within a time interval Δ*t* is small. Therefore, adhesive forces are stronger representing slow, but strong bonds formed by GPIIb-IIIa. At higher and intermediate shear rates, the energy landscape still remains unchanged. However, faster platelets move a larger distance away from each other leading to weaker adhesive forces, which may represent fast, but weak bonds formed by GPIb-vWF. The maximum value of the bond forces in our model based on the calibrated parameters is ≈ 10 *pN*, which is in the range of bond forces measured for GPIb-vWF (catch-slip bonds with maximum lifetime at 20 *pN* [46]), and GPIIb-IIIa-fibrinogen (slip bonds with maximum lifetime at 10 − 20 *pN* [47]) for which the longest bond lifetimes were observed. Further, two activated platelets in our model can only form one bond with each other, whereas each one in the pair can form multiple bonds with the other platelets in its neighborhood, which may result in the distribution of hydrodynamic drag among several bonds. Under pathologic flow conditions where the shear rates are extremely high, the inter-platelet distance *r* is most likely to be ≈ 3*d*, where the same adhesive energy landscape will not be able to slow down or arrest the platelets. Hence, the landscape has to be scaled up with increasing shear rate, which explains the use of $D_{e}^{h}$ in the hyperbolic tangent Eq (10).

Experimental results of Westein *et* *al*. [14] allowed model calibration at medium to high shear rates where the maximum wall shear rate at the apex reaches 8,000 *s*^−1^. One important finding in their work is the marked increase (between two to three fold) of platelet aggregation post-stenosis. Regardless of the molecular mechanisms that can cause such enhanced aggregation at the following edge of a stenosis, we are able to produce similar trends by introducing a platelet activation delay time parameter, *τ*_*act*_. Although there is a physical intrinsic delay in the activation of platelets [28], this parameter is introduced for modeling purposes only; it, too, can be considered as a function of the local blood velocity. Microfluidic experimental results of Li *et* *al*. [33] show a different trend, however, where platelet aggregation initiates at the apex with the highest wall shear rate and then spreads to the inlet and outlet of stenosis. We tested our shear-dependent model against their results, and can achieve similar trends and threshold shear rates at which occlusion occurs.

Numerical modeling of thrombus formation and growth is a challenging problem due to multiscale and multiphysics nature of clotting process, which involves fluid mechanics, cell mechanics, and biochemistry. Diverse studies have addressed this problem on different scales such as cellular, meso and continuum levels (*e*.*g*., refer to [48–52]) whereas attempts have been made to bridge these different scales to model the process at the initial phase of platelet activation and aggregation (*e*.*g*., [53–55]). These studies may be broadly put in three distinct modeling strategies: cellular/sub-cellular modeling of platelet transport and aggregation in whole blood; continuum-based modeling of blood flow treating platelets as Lagrangian particles; and continuum-based modeling of thrombus formation and growth using empirical correlations for platelet deposition rates.

Cellular and multiscale modeling of platelets were used in several studies [28, 30, 48, 51, 53, 54, 56], where the hydrodynamics of blood is resolved and used to model transport of platelets and coagulation enzymes. The kinetic reactions of the coagulation cascade leading to the generation of thrombin and fibrin can be resolved by solving the related advection-diffusion-reaction (ADR) equations. Such detailed models are normally very expensive due to the presence of individual cells and the large set of differential equations related to the biochemistry of coagulation. As a result, they are typically used for mesoscale simulations, and are conducted to explain the relevant microscopic mechanisms and experimental microfluidic observations.

It is possible, however, to reduce the cost of simulations by treating blood and red blood cells as incompressible Newtonian fluid (or non-Newtonian in small arterioles and capillaries), thus leading to continuum fields for blood velocity and pressure and the transport of enzymes, which can be resolved using an Eulerian approach while individual platelets are treated as Lagrangian particles (*e*.*g*., refer to [24, 57]). This numerical approach has the advantage of tracking thousands of platelets forming aggregates at the site of injury and effectively capturing the shape and extent of thrombus. Our proposed model based on FCM falls in this category. FCM provides a flexible platform for *two-way coupling* of platelets (treated as rigid spherical particles) with the background flow. As a result, the thrombus shape modeled by FCM is affected by the local hydrodynamics and fluid stresses. Further, it is possible to introduce porosity to the formed thrombus by adjusting the radius of influence of each particle on the fluid. The major drawback for this kind of approach, however, is the limitation on long-time simulation of large-scale particulate systems for several minutes, which is the physiological time scale of most clotting processes (*e*.*g*., thrombosis following the atherosclerosis plaque rupture or aortic dissection).

Several continuum models treat platelets as concentration fields similar to chemical species that follow specific ADR transport equations [17, 38]. These models could also become expensive depending on the number of species considered, and their outputs are generally more prone to uncertainty due to a large set of input parameters. In a recent work, Mehrabadi *et* *al*. [11] developed a continuum-based model of thrombus formation using empirical correlations for thrombus growth rate as a function of local shear rate using whole blood experiments over a wide range of experimental shear rates. The model has the advantage of predicting thrombus occlusion time with no significant computational cost using a well-trained model by data extracted from different experiments. However, several contributing factors are neglected, including mechanisms of thrombus formation in a low-shear regime, thrombus mechanics, and embolization. These issues can potentially be addressed by introducing platelets as FCM particles, thus forming a hybrid scheme in which the mechanistic behavior of thrombus formation can be resolved while the continuum model accumulates platelets in the thrombus based on empirical correlations until occlusion has been reached.

Including transport equations for different species involved in the coagulation cascade is crucial for accurate predictions of final thrombus shapes, and is straightforward in the current Eulerian-Lagrangian framework. Our numerical simulations of coupled coagulation and platelet aggregation at lower venous flow rates suggest that initiation of coagulation of flowing blood displays a threshold response to shear rate and to the size of the site of injury. This is mainly due to the competition between coagulation reactions at the site of injury and the advection of species from the injury. Similar threshold response was also observed in the *in* *vitro* experiments of Shen *et* *al*. for the whole blood flowing on a surface patch coated with TF [34]. Further, our results show that at lower shear rates platelet aggregation and coagulation can occur independently from each other on two isolated spots at the site of injury leading to the enhanced appearance of fibrin monomers and fibrin deposition. Clinically, stasis and low blood flow are considered risk factors for deep vein thrombosis. As shear rate increases in blood flow through arterioles, advective effects become more dominant, which could eliminate thrombin production on the subendothelium. Therefore, the role of heterogeneous coagulation reactions on the surface of adhered platelets would become more crucial to the progression of thrombosis, and must be included in future numerical models.

One of our goals is to improve our understanding of the effects of hemodynamics on the initiation and development of intramural thrombus within a false lumen caused by an aortic dissection. Besides their greater complexities in geometry and flow conditions compared to the microscopic systems considered in this study, the size of aortic dissections are rather large. Therefore, simulations may require hundreds of thousands of FCM particles to represent platelets. Even the computational cost for such lower-fidelity simulations in large domains could become restrictive, and may require additional modeling strategies that will be addressed in future work.

### Conclusion

We developed an Eulerian-Lagrangian model to predict thrombus shape and growth, where motions of Lagrangian platelets are coupled with the background blood flow using a force coupling method. Further, platelet adhesion to the site of injury and to each other is modeled by a shear-dependent Morse potential, which is calibrated with experimental data for different shear conditions. Our simulation results show good agreement with experiments for a wide range of shear rates, thus suggesting that the proposed method is suitable for modeling venous thrombosis and embolization as well as thrombosis in arteries.

## Supporting Information

S1 Appendix The coagulation cascade. As mentioned in the main text, we use the coagulation model from Anand *et* *al*. [18], where both the extrinsic or TF pathway and intrinsic or contact pathway are considered. The intrinsic pathway is initiated when XII is activated to XIIa. Subsequently, XIIa activates the zymogen XI to its active enzyme form XIa, which further activates IX to IXa in the presence of Ca^2+^. The role of intrinsic pathway on the propagation of coagulation under flow conditions is not quite known, but has been included here for the sake of completeness of the biochemical model (with the exception of factor XII). The list of reactants and their normal initial concentration along with their diffusion coefficients in blood plasma are given in S1 Table. The equations governing the generation and depletion of the species (*S*_*i*_ in Eq (11)) are formulated based on experimental data for the reaction kinetics, and are listed in S2 Table. The kinetic constants, also obtained from experimental data, are given in the table’s caption. Further, concentrations of two other chemical species tenase (Z) and prothrombinase (W) are computed through the relations [Z] = [VIIIa][IXa]/*K*_*dZ*_ and [W] = [Va][Xa]/*K*_*dW*_, respectively [18]. At the site of injury, we assume that the subendothelium-bound TF-VIIa complex drives the extrinsic pathway of the coagulation cascade through the subendothelium reactions that are represented by Neumann boundary conditions in the form of −*D*_*j*_∂*c*_*j*_/∂*n* = *B*_*j*_. Surface reactions *B*_*j*_ along with their kinetic constants are given in S3 Table. Further, [TF-VIIa]^*W*^ is prescribed at the wall as a decreasing cubic function of fibrin concentration given the fact that fibrin deposition leads to fewer subendothelium binding sites available for the complex. The initial value of the concentration is considered to be in the physiologic range [TF-VIIa]^0^ ≈ 1 (*nM*) [16].
(TEX)

S1 TableList of reactants in the coagulation cascade along with their initial concentrations $c_{i}^{0}$ and diffusion constants *D*_*i*_ [18].(TEX)

S2 TableReaction equations for source terms.Kinetic constants are given as follows: *k*_9_ = 11 *min*^−1^, *K*_9*M*_ = 160 *nM*, *h*_9_ = 0.0162 *nM*^−1^ *min*^−1^, *k*_8_ = 194.4 *min*^−1^, *K*_8*M*_ = 112,000 *nM*, *h*_8_ = 0.222 *min*^−1^, *h*_*C*8_ = 10.2 *min*^−1^, *H*_*C*8*M*_ = 14.6 *nM*, *k*_5_ = 27.0 *min*^−1^, *K*_5*M*_ = 140.5 *nM*, *h*_5_ = 0.17 *min*^−1^, *h*_*C*5_ = 10.2 *min*^−1^, *H*_*C*5*M*_ = 14.6 *nM*, *k*_10_ = 2391 *min*^−1^, *K*_10*M*_ = 160 *nM*, *h*_10_ = 0.347 *nM*^−1^ *min*^−1^, *h*_*TFPI*_ = 0.48 *nM*^−1^ *min*^−1^, *k*_2_ = 1344 *min*^−1^, *K*_2*M*_ = 1060 *nM*, *h*_2_ = 0.714 *nM*^−1^ *min*^−1^, *k*_1_ = 3540 *min*^−1^, *K*_1*M*_ = 3160 *nM*, *h*_1_ = 1500 *min*^−1^, *H*_1*M*_ = 250,000 *nM*, *k*_11_ = 0.0078 *min*^−1^, *K*_11*M*_ = 50 *nM*, $h_{11}^{A3}=1.6\times 10^{-3}\;nM^{-1}min^{-1}$, $h_{11}^{L1}=1.3\times 10^{-5}\;nM^{-1}min^{-1}$, *k*_*PC*_ = 39 *min*^−1^, *K*_*PCM*_ = 3190 *nM*, *h*_*PC*_ = 6.6 × 10^−7^ *nM*^−1^ *min*^−1^, *K*_*dZ*_ = 0.56 *nM*, *K*_*dW*_ = 0.1 *nM*.(TEX)

S3 Table Flux boundary conditions imposed at the injured vessel wall. The kinetic constants are given as *k*_7,9_ = 32.4 *min*^−1^, *K*_7,9*M*_ = 24 *nM*, *k*_7,10_ = 103.0 *min*^−1^, *K*_7,10*M*_ = 240 *nM*.
(TEX)
